# Assessment of Epidemiology Capacity in State Health Departments — United States, 2013

**Published:** 2015-04-17

**Authors:** James L. Hadler, Rebecca Lampkins, Jennifer Lemmings, Meredith Lichtenstein, Monica Huang, Jeffrey Engel

**Affiliations:** 1 Yale University School of Public Health, New Haven, Connecticut; 2 Council of State and Territorial Epidemiologists, Atlanta, Georgia

Since 2001, the Council of State and Territorial Epidemiologists (CSTE) periodically has conducted a standardized national assessment of state health departments’ core epidemiology capacity (1–4). During August–September 2013, CSTE sent a web-based questionnaire to state epidemiologists in the 50 states and the District of Columbia. The questionnaire inquired into workforce capacity and technology advancements to support public health surveillance. Measures of capacity included the total number of epidemiologists, a self-assessment of the state’s ability to carry out four of the 10 essential public health services* most relevant to epidemiologists, and program-specific epidemiology capacity. This report summarizes the results, which indicated that in 2013, most of these measures were at their highest level since assessments began in 2001, including the number of epidemiologists, the percentage of state health departments with substantial-to-full (>50%) capacity for three of the 10 essential public health services, and the percentage with substantial-to-full epidemiology capacity for eight of 10 program areas. However, >50% of states reported minimal-to-no (<25%) epidemiology capacity for four of 10 program areas, including occupational health (55%), oral health (59%), substance abuse (73%), and mental health (80%). Federal, state, and local agencies should work together to develop a strategy to address continued outstanding gaps in epidemiology capacity.

The main objectives of the periodic CSTE epidemiology capacity assessments (ECA) are to count and characterize the state-employed epidemiologist workforce and to measure current core epidemiology capacity. CSTE standardized assessments began in 2001 (1) and were conducted in 2004, 2006, 2009 (supplemented by a rapid enumeration in 2010), and 2013 (2–4). Some of the information sought by the assessments relate to the four most epidemiology-related essential public health services. These include 1) monitoring health status to identify and solve community health problems; 2) diagnosing and investigating health problems and health hazards in the community; 3) evaluating effectiveness, accessibility, and quality of personal and population-based health services; and 4) conducting and evaluating research for new insights and innovative solutions to health problems. The first three assessments evaluated capacity in eight program areas: infectious diseases, bioterrorism/emergency response, chronic disease, maternal and child health, environmental health, injury, occupational health, and oral health. In 2009, questions were added to assess substance abuse epidemiology capacity and implementation of selected surveillance-related technology advancements, and in 2013, to assess mental health epidemiology capacity.

After pilot testing, CSTE made the 2013 ECA questionnaire available online to all states during August 23–September 30, 2013. The state epidemiologist was designated the key informant, and lead epidemiologists added information for program-specific questions. The state epidemiologist also distributed a worksheet on training experience and program areas of work to each individual enumerated epidemiologist. All 50 states and the District of Columbia participated. An epidemiologist was defined as any person who, regardless of job title, performed functions consistent with the generally accepted definition† (5). Part-time positions and full-time positions in which epidemiologists did only part-time epidemiology work were reported as fractions of full-time equivalents. The state epidemiologist was asked whether the state health department had adequate epidemiology capacity to provide the services and to estimate the extent to which their department met the activity for the essential public health service.§ Estimates were categorized as follows: full capacity was defined as having 100% of the activity, knowledge, or resources described within the question; almost full capacity was defined as having 75%–99%; substantial capacity was defined as having 50%–74%; partial capacity was defined as having 25%–49%; minimal capacity was defined as having some but <25%; and no capacity was defined as having zero. For each program area, the extent of epidemiology and surveillance capacity was assessed by using the same scale.¶ The state epidemiologist also was asked to estimate the ideal number of epidemiologists needed to meet epidemiology and surveillance capacity for each program area fully. Population estimates from the U.S. Census for 2010 were used as denominators.

In 2013, a total of 2,752 epidemiologists worked for the 51 jurisdictions, a ratio of 0.87 epidemiologists per 100,000 population (state median: 1.04; range: 0.19–5.72), an increase of 25% from the 2,193 epidemiologists reported in 2009 and an increase of 10% from the previous high of 2,498 in 2004. Among respondents, 42 (82%) reported substantial-to-full capacity to monitor health status and solve community health problems, and 46 (90%) reported the same capacity to diagnose and investigate health problems and hazards in the community. In contrast, only 18 (35%) reported substantial-to-full capacity to evaluate effectiveness, accessibility, and quality of personal and population-based health services, and 15 (29%) reported the same capacity to conduct research for new insights and innovative solutions to health problems. Except for the evaluation EPHS, the percentage of states reporting substantial-to-full capacity was the highest to date (Figure 1).

When compared with results from the 51 jurisdictions from 2004 through 2009, all program areas except substance abuse showed increases in substantial-to-full capacity to their highest levels to date: infectious diseases (98%), bioterrorism/emergency response (82%), maternal-child health (73%), chronic disease (66%), environmental health (49%), injury (45%), occupational health (20%) and oral health (25%) (Figure 2). For four program areas, the majority reported minimal-to-no capacity: occupational health (28 [55%]), oral health (30 [59%]), substance abuse (37 [73%]) and mental health (41 [80%]). On the basis of responses about needs, and assuming that nonresponse meant no additional need, adding 1,374 epidemiologists (a 50% increase to 1.31 epidemiologists per 100,000 population nationally) is needed to achieve ideal epidemiology and surveillance capacity in all program areas.

The assessment of technology capacity to support surveillance showed that 33 states (67%) had fully automated electronic laboratory reporting, 15 (29%) used automated cluster detection software, and fewer than half routinely geocoded reportable disease data (19 [37%]), births (25 [49%]) or deaths (24 [47%]), all improvements since 2009 (Table).

Among 2,752 enumerated epidemiologists, 1,586 (58%) completed worksheets describing their level of formal epidemiology training. Compared with the 2009 ECA, a nonstatistically significant slightly higher percentage had a master’s or higher level degree in epidemiology (59% versus 56%) and a lower percentage had no formal training or academic coursework in epidemiology (12% versus 13%). State epidemiologists reported that 260 (11%) staff epidemiologists with advanced degrees retired or left their job during 2012; 18% of the current workforce anticipates leaving within 5 years.

## Discussion

Epidemiology capacity is essential for detection, control, and prevention of major public health problems. Epidemiology provides information needed to perform four of the 10 essential public health services. *Healthy People 2020* calls for the United States to increase the proportion of tribal, state, and local public health agencies that provide or assure comprehensive epidemiology services to support essential public health services (6). CSTE’s periodic ECA is the major data source for monitoring progress toward achieving this objective.

The 2013 ECA revealed the highest levels yet in most measured aspects of state-level epidemiology capacity. The factors leading to the improvements are unclear but were noted in the late 2010 rapid assessment which enumerated a 13% increase in state-level epidemiologists from the nadir in 2009 (7). The increase coincided with federal stimulus funding. Since then, the economy has strengthened and stimulus-supported initiatives, e.g., monitoring health care-associated infections, have continued.

The 2013 ECA identified substantial ongoing gaps in epidemiology capacity. These included low levels of epidemiology capacity for occupational and oral health, very low levels of health department involvement in substance abuse and mental health surveillance and epidemiology, and continued lack of key technology capacity and capacity for evaluating effectiveness of prevention efforts and for conducting research for new insights and innovative solutions in many states. Without public health involvement, the contribution of these areas to the overall public health is not well measured or monitored, and primary and secondary prevention efforts are less likely to be implemented and evaluated at the population level. Without technology capacity to conduct state-of-the-art surveillance (e.g., automated electronic laboratory-based reporting, cluster-detection software, and geocoding), reporting will be less timely and less complete, the ability to detect outbreaks rapidly and expand laboratory-based surveillance will be reduced, and less use will be made of geographic information systems to describe and respond to inequities in health better. The fewer the number of states with capacity to evaluate any prevention efforts or to conduct research for new insights and innovative solutions to public health problems, the smaller our national capacity to explore new ideas and identify successful ones.

What is already known on this topic?

Data on state-level epidemiology capacity from surveys conducted by the Council of State and Territorial Epidemiologists (CSTE) since 2001 indicate that capacity in many areas previously peaked in 2004, a time of peak federal funding for public health preparedness, and then diminished to especially low levels by 2009.

What is added by this report?

Data from the most recent CSTE survey indicate that overall state-level epidemiology capacity and the epidemiology capacity in many program areas has increased markedly since 2009. The number of epidemiologists, the percentage of state health departments with substantial-to-full capacity for three essential public health services, and the percentage with substantial-to-full epidemiology capacity for eight of 10 program areas were at their highest level since assessments began in 2001. However, >50% of states reported minimal-to-no epidemiology capacity in occupational health, oral health, substance abuse, and mental health. Most health departments still lack critical technology capacity.

What are the implications for public health practice?

State, federal, and local agencies should work together to address underdeveloped surveillance and epidemiology capacity, particularly in mental health, substance abuse, oral health, and occupational health by reaching a consensus on optimal levels and developing a strategy to achieve them.

The findings of this report are subject to at least two limitations. First, the 2013 assessment only measured epidemiology capacity of state health departments. Approximately one third of all public health epidemiology capacity located in states is in local health departments (7). Second, the methods used by respondents to estimate their capacity to perform the essential services of public health, program-specific epidemiology capacity, and the numbers needed to reach ideal capacity were self-reported.

State, federal, and local agencies should work together to address underdeveloped surveillance and epidemiology capacity, particularly in mental health, substance abuse, oral health, and occupational health by reaching a consensus on optimal levels and developing a strategy to achieve them.

## Figures and Tables

**FIGURE 1:**
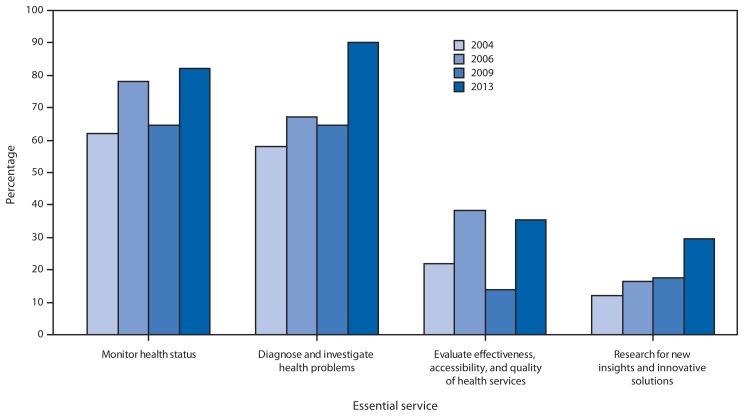
Percentage of state health departments reporting substantial-to-full (>50%) capacity in four essential services of public health — Council of State and Territorial Epidemiologists Epidemiology Capacity Assessment, United States,* 2004, 2006, 2009, and 2013 * 50 states and the District of Columbia.

**FIGURE 2:**
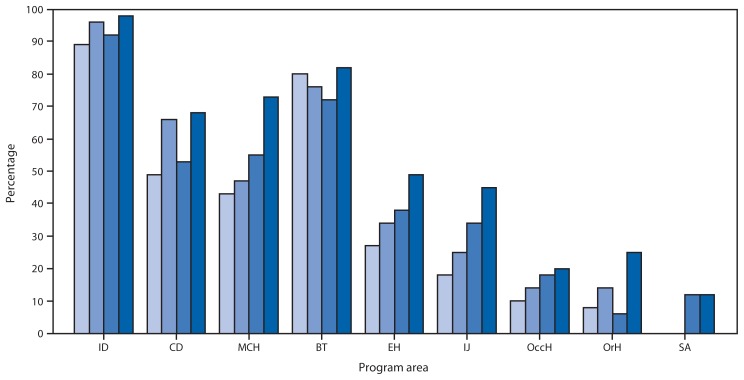
Percentage of state health departments reporting substantial-to-full (>50%) capacity in epidemiology and surveillance programs, by program area — Council of State and Territorial Epidemiologists Epidemiology Capacity Assessment, United States,* 2004, 2006, 2009, and 2013 **Abbreviations:** ID = infectious diseases, CD = chronic diseases, MCH = maternal and child health, BT = bioterrorism and emergency response, EH = environmental health, IJ = injury, OccH = occupational health, OrH = oral health, SA = substance abuse. * 50 states and the District of Columbia.

**TABLE:** Number and percentage of state health departments with selected technology capacities to support epidemiology and surveillance — Council of state and Territorial Epidemiologists Epidemiology Capacity Assessment, United States,* 2009 and 2013

Technology capacity	2009		2013	
	
	No.	(%)	No.	(%)
Automated ELR	27	(53)	33	(66)
Expanded no. reportable conditions due to ELR (among those with ELR)	10	(37)	13	(39)
Cluster-detection software	12	(24)	15	(29)
Syndromic surveillance	–	–	40	(78)
Outbreak-management system	16	(31)	23	(45)
Geocode births	20	(39)	25	(49)
Geocode deaths	21	(41)	24	(47)
Geocode all reportable diseases	15	(29)	19	(37)
Geocode some reportable diseases	28	(55)	31	(61)

**Abbreviation:** ELR = electronic laboratory reporting

* 50 states and District of Columbia. All questions responded to by 51 jurisdictions except automated ELR had 50 respondents in 2013. Syndromic surveillance capacity not asked in 2009.

## Footnotes

* Additional information about the 10 essential public health services is available at http://www.cdc.gov/nphpsp/essentialservices.html.

† “An investigator who studies the occurrence of disease or other health-related conditions or events in defined populations. The control of disease in populations is often also considered to be a task for the epidemiologist, especially in speaking of certain specialized fields such as malaria epidemiology. Epidemiologists may study disease in populations of animals and plants, as well as among human populations.” Source: Last JM, Spasoff RA, Harris SS, Thuriaux MC, eds. A dictionary of epidemiology. 4th ed. New York, NY: Oxford University Press; 2001.

§ The question asked was, “Does your state health department have adequate epidemiologic capacity to provide the following four essential public health services?”

¶ The question asked was, “What is the extent of the epidemiology and surveillance capacity in the following program areas in your state health department? If needed, please seek the guidance of other state health department staff within program specific areas when completing this question.”
